# Investigation and Optimization of the C-ANN Structure in Predicting the Compressive Strength of Foamed Concrete

**DOI:** 10.3390/ma13051072

**Published:** 2020-02-28

**Authors:** Dong Van Dao, Hai-Bang Ly, Huong-Lan Thi Vu, Tien-Thinh Le, Binh Thai Pham

**Affiliations:** 1 University of Transport Technology, Hanoi 100000, Vietnam; banglh@utt.edu.vn (H.-B.L.); lanvth@utt.edu.vn (H.-L.T.V.); binhpt@utt.edu.vn (B.T.P.); 2 Institute of Research and Development, Duy Tan University, Da Nang 550000, Vietnam

**Keywords:** Compressive Strength, Foamed Concrete, Artificial Neural Network, Monte Carlo simulations

## Abstract

Development of Foamed Concrete (FC) and incessant increases in fabrication technology have paved the way for many promising civil engineering applications. Nevertheless, the design of FC requires a large number of experiments to determine the appropriate Compressive Strength (CS). Employment of machine learning algorithms to take advantage of the existing experiments database has been attempted, but model performance can still be improved. In this study, the performance of an Artificial Neural Network (ANN) was fully analyzed to predict the 28 days CS of FC. Monte Carlo simulations (MCS) were used to statistically analyze the convergence of the modeled results under the effect of random sampling strategies and the network structures selected. Various statistical measures such as Coefficient of Determination (R^2^), Mean Absolute Error (MAE), and Root Mean Squared Error (RMSE) were used for validation of model performance. The results show that ANN is a highly efficient predictor of the CS of FC, achieving a maximum R^2^ value of 0.976 on the training part and an R^2^ of 0.972 on the testing part, using the optimized C-ANN-[3–4–5–1] structure, which compares with previous published studies. In addition, a sensitivity analysis using Partial Dependence Plots (PDP) over 1000 MCS was also performed to interpret the relationship between the input parameters and 28 days CS of FC. Dry density was found as the variable with the highest impact to predict the CS of FC. The results presented could facilitate and enhance the use of C-ANN in other civil engineering-related problems.

## 1. Introduction

Over the past decades, large-scale, super-tall, or mega-tall buildings have been increasingly constructed over the world [1,2]. This fact raises a number of fundamental engineering problems, crucial to be solved, such as self-weight, large foundation sizes, and earthquake resistance [3,4]. Apart from the structural problems, the weight of conventional concrete is also a major drawback from a material point of view [5]. Foamed Concrete (FC) appears as a potential replacement of conventional concrete to be used in specific engineering applications. Since the early 1920s, when the Swedish architect Johan Eriksson was granted the patent on FC, many further inventions have been developed and applied in practice by experts in America, Japan, and Europe [6]. Lightweight concrete, known as cellular concrete or FC, is a recently developed concrete with many advantages [7,8]. Cellular concrete is made by generating air bubbles in the cement paste or mortar. These bubbles have diameters ranging from 0.1 to 1 mm [9,10] and air is usually contained within 50% of the volume [11]. The presence of bubbles reduces the specific gravity of FC to between 400 and 1600 kg/m^3^ [3,12,13]. Many researches have pointed out that the outstanding advantages of FC is lightweight, thermal insulation, sound insulation, and void filling [6,11]. Moreover, FC can be produced at low cost, it is easy to construct, and benefits from being a low waste, eco-friendly material [14]. Therefore, the use of FC is increasing in the construction industry [3], for example, as thermal insulation materials for walls, sound barriers, fire walls or backfill structures, foundations, and panels for construction [11]. Furthermore, FC’s low elastic modulus also eases its use in roads and tunnels, for instance, embedding of large diameter pipelines as roadbed or cushion materials to homogeneously disperse the stress from external loadings [6]. On the contrary, several problems still exist that require further investigations, which can be listed as poor stability, low strength, higher shrinkage, easy cracking, and water absorption. In particular, the Compressive Strength (CS) of FC is often low and it is often difficult to reach 25 MPa in certain cases [12,15]. The CS of FC also depends on the composition or mixture from the constituents. These points limit the application of FC in structural engineering [16,17]. As a consequence, assessing the CS of FC is crucial and becoming an essential task for researchers.

In the literature, various approaches to forecast the CS of FC have been investigated [3,6,8,9,10,12,14,15,16,17]. Numerous studies have also reported that the composition of the substances structure and their proportions greatly affect the CS of FC [18,19,20]. For example, in the work of Asadzadeh and Khoshbayan [21], to optimize various types of concrete characteristics, design-of-experiments-based statistical methods have been utilized. Response Surface Methodology (RSM) and design-expert technology have been used to create a relationship between the input factors and the mechanical responses of fly-ash lightweight concrete [22]. However, for the crushing intensity, with a standard deviation of 3.99, the coefficient of determination (R^2^) is only achieved at an R^2^ of 0.60. In different approaches proposed by Bing et al. [23] or Liu et al. [24], the experimental studies have been carried out with a number of 16 different types of FC, with 21 cubes of 100 mm and prisms of 100x100x515 mm^3^ casted and used to investigate the development of CS over time, as well as the effect of foam volume content [23]. Regardless of these efforts, the experimental cost and time consumption are major obstacles of this approach. On the other hand, empirical equations using experimental data could be an alternative approach to estimate the CS of FC. Nevertheless, the range of input conditions is the main reason limiting the predicted CS of FC using these equations [25]. In addition, such semi-analytical equations require several constants that are not easy to obtain and highly depend on the complex relationships between the mixture constituents and the CS of FC [18,19,20,25,26]. Therefore, the development of an advanced numerical tool for prediction of the CS of FC is essential.

Over the past decades, machine learning (ML) models as a brand of artificial intelligence (AI) techniques have been widely used in civil engineering. Many complex problems related to structural engineering [27,28,29], material sciences [30,31,32,33], geotechnical engineering [34,35,36,37,38,39], and earth sciences [40,41,42,43,44,45,46] have been successfully resolved. The prediction of the mechanical properties of FC has also been the subject of many studies in the literature. In the work of Nguyen et al. [25], a deep neural network (DNN) model has been used to predict the CS of FC. The authors collected 177 experimental results available in the literature and obtained satisfactory correlation results with the predicted output of DNN. Besides, the gene expression programming (GEP) algorithm, an extension of the genetic algorithm (GA) and genetic programming (GP), has been applied in the work of Kiani et al. [9]. In particular, it is impossible not to mention the work of Abd et al. [47] when using Support Vector Machines (SVM) applied to 150 results from laboratory experiments. SVM using a radial basis function was proven more accurate than other functions and traditional regression functions [47]. Besides, various ML algorithms (MLR, ANN, SVR, MARS, and MARS–WCA) have been used to predict the CS of FC at different testing ages [26]. It is thus confirmed that ML algorithms are powerful numerical tools that can account for complex relationships between mixture components and help in optimizing the mixture to achieve the targeted mechanical properties, such as the CS of FC.

In this study, the performance of a Conventional Artificial Neural Network (C-ANN) algorithm was investigated to predict the 28 days CS of FC. Although ANN is one of the most effective ML algorithms, its performance depends significantly on the selection of network structure. Thus, this study also focuses on the investigation and optimization of the C-ANN structure for better prediction of the CS of FC. To achieve this goal, previously published experimental data in the literature were gathered and randomly divided into two subsets: the training dataset (70% of data) and the testing part (30% of data). Monte Carlo simulations (MCS) were used to verify the convergence of the modeled results. In addition, Partial Dependence Plots (PDP) over 1000 MCS were also produced to interpret the relationship between input parameters and 28-day CS of FC. Various statistical measures such as Coefficient of Determination (R^2^), Mean Absolute Error (MAE), and Root Mean Squared Error (RMSE) were used for validation of model performance. 

## 2. Statement of the Novelty and Significance

The 28-days CS of FC is a key value in structural engineering, controlling the quality of construction works. While various surrogate, optimized, and hybrid ML algorithms have been used, it has been proven that a C-ANN model could still accurately predict the 28-days CS of FS, which depends significantly on the selection of the appropriate network structure. Limited efforts to find out a suitable C-ANN structure have been reported in the literature [48,49], but no systematic study has accounted for random sampling of the dataset. Therefore, the current study contributes to fill these research gaps through the following points: (i) an improved prediction of the CS of FC was achieved using C-ANN algorithm; (ii) MCS was used, for the first time, to determine the most effective C-ANN structure in the presence of random dataset splitting; (iii) a comparison with existing published results was conducted to confirm the accuracy of C-ANN; (iv) a sensitivity analysis was performed using PDP to reveal the relationships between the input variables and the 28-days CS of FC; and (v) three equations aiming to interpret the dependence of the 28-days CS of FC on dry density, water-to-cement, and sand-to-cement ratios were derived from PDP investigation.

## 3. Materials and Methods

### 3.1. Data Used

**Figure 1:**
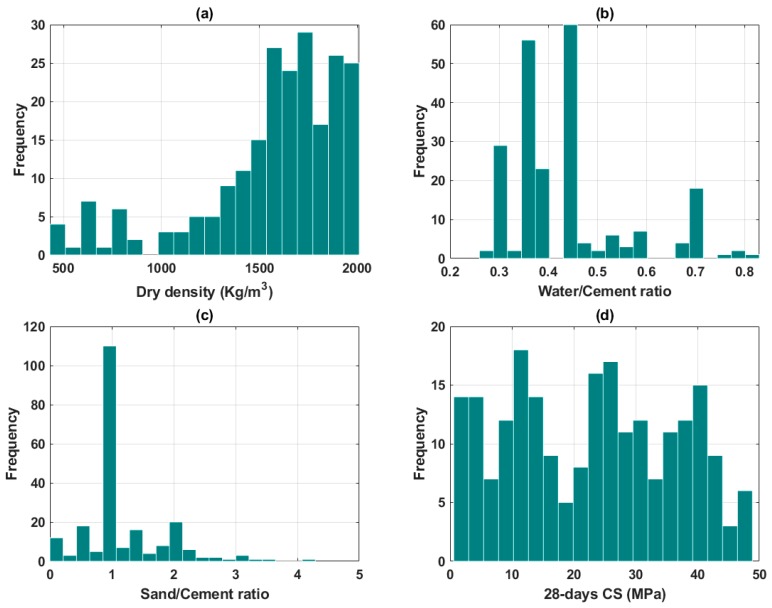
Distribution histograms of (**a**) dry density; (**b**) water/cement ratio; (**c**) sand/cement ratio, and (**d**) 28-days Compressive Strength (CS) of Foamed Concrete (FC).

In this study, 220 data points of experiments on FC collected from the literature (Table 1) were used, including dry density (D), water/cement ratio (W/C), and sand/cement ratio (S/C) as input variables, and the CS at 28 days as an output variable. Table 1 summarizes the information of the literature constituting the database, including the number of data points collected. Besides, a summary of the statistical analysis of the three inputs and the CS as output of this study is presented in Table 2. The distribution histograms of dry density (D), water/cement ratio (W/C), sand/cement ratio (S/C), and the CS is displayed in Figure 1.

### 3.2. Methods Used

#### 3.2.1. Conventional Artificial Neural Network (C-ANN)

In the past decades, along with the intense development of computer science, AI techniques have been used to a greater extent in various areas of expertise [51]. C-ANN is the most common AI algorithm used in scientific research, especially in civil engineering [52]. C-ANN is an information processing computing system designed based on the operation of the human brain [35,53,54,55,56]. The very first study of ANN was proposed by McCulloch and Pitts [57] in the early 1940s. In fact, C-ANN is made up of multiple nodes and links that bind these nodes, and a weight which is capable of learning is associated with each link. They learn by changing the value of the weights. The training algorithm’s objective is to reduce the mean square error (MSE) between the target and the predicted outputs. 

The advantages of C-ANN could be various but the most important point of ANN comes from the ability to handle highly nonlinear problems [58]. Thus, complex interactions, relationships between inputs and outputs, can be modeled without knowing the correlation between these variables [58]. Thanks to these advantages C-ANN is applied in many different fields, such as basic sciences, construction, health, and environment. The implementations of C-ANN in civil engineering are promising and many important results have been achieved over the past years [52]. In the study of Nehdi et al. [59], C-ANN was used to predict the cellular concrete output. The investigation results show that compared to existing parametric methods, C-ANN can much more accurately predict several properties of concrete, such as production yield, foamed density, un-foamed density, and the CS of cellular concrete mixtures. In another study, from 150 empirical results, Paulson et al. [60] used C-ANN to conduct a comprehensive survey to optimize the level of cement replacement with silica fume. This study pointed out that it was expected that CS and the values were similar to the experimental results, so it concluded that instead of conducting experiments, C-ANN can be used to predict the CS for various values of input [60].

#### 3.2.2. Quality Assessment of Results

In the present study, the coefficient of determination (R^2^), Mean Absolute Error (MAE), and Root Mean Squared Error (RMSE) are used as measures to evaluate the developed ML algorithms. Precisely, R^2^ values allow to identify the statistical relationship between the experimental results and predicted values. It yields a value between 0 and 1, where 0 is no correlation and 1 is total correlation [61]. In the cases of RMSE and MAE, which have the same units as the quantity being estimated, lower values of RMSE and MAE basically indicate a high accuracy of prediction output using the ML models [29,32,62,63]. Values of R, RMSE, and MAE are estimated using the following equations [31,61,64,65]:

(1) $$\mathrm{MAE}=\frac{\sum_{i=1}^{n}\left|p_{i}-v_{i}\right|}{n}$$

(2) $$\mathrm{RMSE}\;=\;\sqrt{\sum_{i=1}^{n}\frac{{\left(p_{i}-v_{i}\right)}^{2}}{n}}$$

(3) $$R^{2}=\frac{\sum_{i=1}^{n}(p_{i}-q)(v_{i}-\underline{v})}{\sqrt{\sum_{i=1}^{n}(p_{i}-q{)}^{2}}\sum_{i=1}^{n}(v_{i}-\underline{v}{)}^{2}}$$

where *n* is defined as the number of input data, *p_i_* and *v_i_* denoted the actual and predicted CS values, respectively, and $\underline{v}$ is the mean value of the predicted CS by C-ANN.

#### 3.2.3. Monte Carlo Simulations (MCS)

The performance of any ML algorithm is highly depended on the way that the training dataset is constructed—that is, the data sampling method. Data sampling is a kind of statistical method used to select observations from the database whose main objective is to investigate a selected parameter. In applied machine learning, data sampling is usually referred to as the propagation of variability of inputs on the predicted output. The real performance of ML algorithms could only be accepted only if the prediction errors are low even with the presence of variability in the dataset. In order to generate such variability, MCS is used in this study. The Monte Carlo method is one of the well-known techniques to propagate input variability on the output results [63,66,67,68,69]. The Monte Carlo method is notably powerful and efficient for evaluating the performance of ML models [34,70]. Moreover, Monte Carlo allows massively parallel computing to be conducted, making it an effective strategy to reduce simulation time [71,72,73,74]. The main idea of the MCS is to use random sampling to generate as many realizations as possible in the input space. The next step is dedicated to the calculation of the output through ML models [75,76]. Finally, the obtained results of performance could be assessed using several statistical criteria. In this work, the statistical convergence of MCS is examined using the equation as follows [62,70,77,78]:

(4) $$f_{M}\left(M\right)=\frac{1}{\underline{K}}\frac{1}{M}\sum_{i=1}^{M}K_{i},$$

where $\underline{K}$ is the mean value of the considered random variable K and M is the number of Monte Carlo runs. This convergence function provides efficient information related to the computational time, reliable results for further statistical analysis.

### 3.3. Methodological flow chart

The methodology flowchart of the present study included five main steps, as follows:

Step 1: Preparation and pre-processing of the database, including 220 data units collected from the available literature. The three input variables were dry density, water-to-cement, and sand-to-cement ratios, and the output was the CS of FC. The histograms of the distribution of the inputs and outputs were next presented. Before further processing, the database was scaled in the (0,1) range to reduce numerical bias, as in common ML problems.

Step 2: To analyze the convergence of the C-ANN under random sampling, a number of 1000 of simulations was conducted for each combination of C-ANN. The number of neurons in the first hidden layer of the C-ANN structure was varied from 1 to 20, whereas those of the second hidden layer ranged from 0 to 20. The value of 0 is denoted with the case using only one hidden layer. A total of 420 structures was constructed using one and two hidden layers, making a total of 420,000 simulations (21 neurons in the first hidden layer x 20 neurons in the second hidden layer x 1000 simulations). It is worth noting that the exact values of the error criteria are not presented in this step. It is dedicated only for the convergence analysis of the two typical C-ANN structures.

Step 3: To determine the optimized structure of C-ANN, various structures of C-ANN were developed and validated using R^2^, RMSE, and MAE. The optimized C-ANN structure was selected based on the maximum values of R^2^ and minimum values of RMSE and MAE. 

Step 4: Using the optimal C-ANN structure, a detailed analysis of robustness of C-ANN algorithm was carried out and presented.

Step 5: Interpretation of the relationship and dependence of the 28-days CS with the input factors (dry density, water/cement ratio, and sand/cement ratio) using C-ANN was presented using PDP.

## 4. Results 

### 4.1. Convergence of C-ANN under Random Sampling Effect 

**Figure 2:**
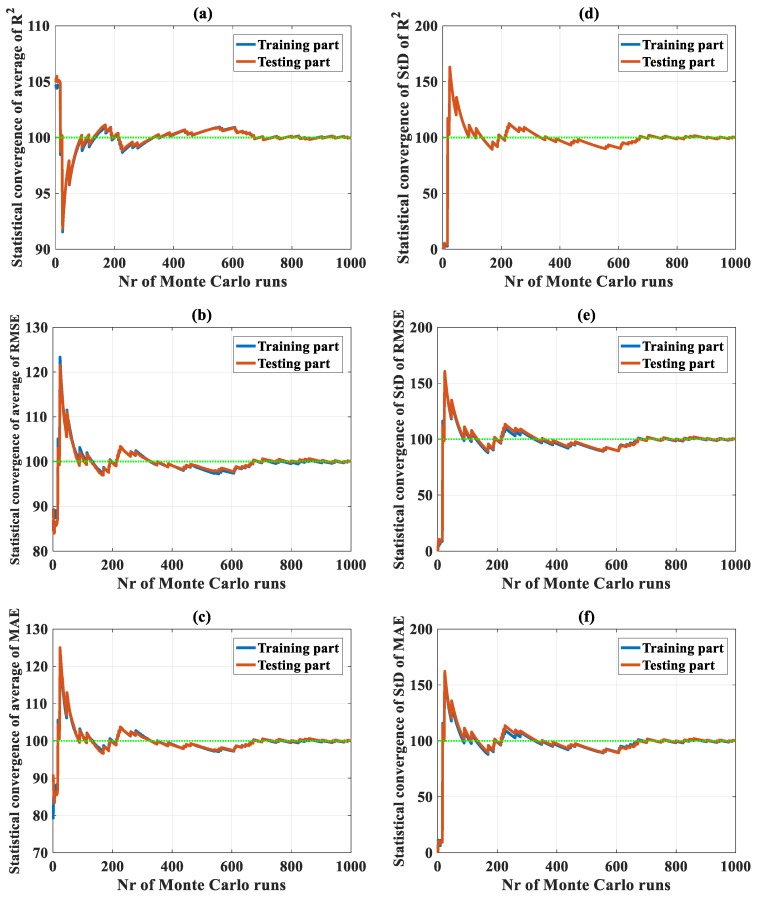
Convergence of random sampling in the case of using C-ANN-[3–1–1–1] for the average values of (**a**) R2, (**b**) RMSE, and (**c**) MAE; for the standard deviation (SD) values of (**d**) R^2^, (**e**) RMSE, and (**f**) MAE.

**Figure 3:**
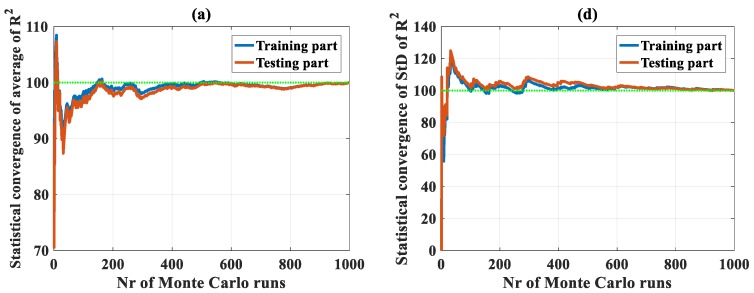

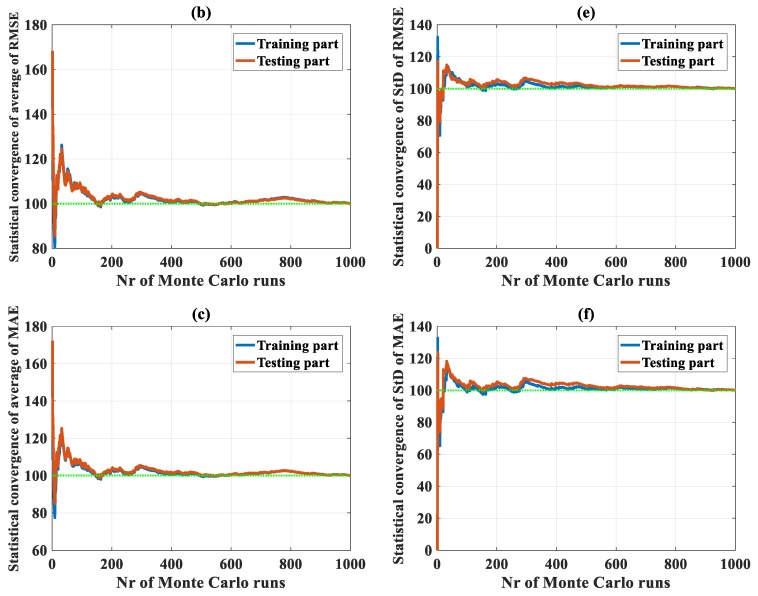
Convergence of random sampling in the case of using C-ANN-[3–20–20–1] for the average values of (**a**) R2, (**b**) RMSE, and (**c**) MAE; for the standard deviation (SD) values of (**d**) R^2^, (**e**) RMSE, and (**f**) MAE.

Convergence of C-ANN under the random sampling effect was analyzed with a total of 420,000 simulations for 420 C-ANN structure cases. However, the convergence results of only two typical C-ANN structures (C-ANN-[3–1–1–1] and C-ANN-[3–20–20–1]) are presented in Figure 2; other cases are not shown. It is observed that the number of 1000 simulations were satisfactory for both the training and testing datasets for each case (Figure 2). With respect to the values of R^2^ using C-ANN-[3–1–1–1], a fluctuation of 1% around the mean value was reached from 100 simulations (Figure 2a), whereas with the same number of runs, RMSE and MAE fluctuated around 4% of the average values (Figure 2b,c). The predicted results stabilized around the mean values (green discontinuous lines) when the number of MCS was 700. The standard deviation error of R^2^, RMSE, and MAE exhibited similar trends, but getting close to the average values at 700 runs. Considering the structure C-ANN-[3–20–20–1] with the results plotted in Figure 3, the fluctuation in terms of R^2^ seemed more important than the previous case, i.e., from 70% to 110% compared with a range of 90% to 105%. The 1% level of fluctuation was reached from above 800 simulations, compared with 100 runs in the previous case. An identical number of simulations was also required for the case of RMSE and MAE, as well as a more important fluctuation that was observed in contrast with the C-ANN-[3–1–1–1] structure. Nevertheless, the standard deviation of the three error criteria converged toward a value at about 500 runs (Figure 3d,e,f), smaller than the results obtained by C-ANN-[3–1–1–1].

It can be observed that the two extracted typical C-ANN structures converged toward the statistical results. Thus, all the remaining C-ANN structures, with the number of neurons in the two hidden layers varied from 1 to 20, could also converge toward the simulation results. Thus, it can be stated that the C-ANN converged under the random sampling effect of the data used in this study.

### 4.2. Optimization of C-ANN Architecture

**Figure 4:**
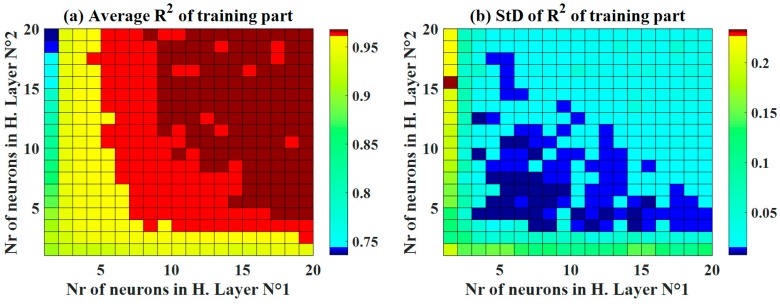

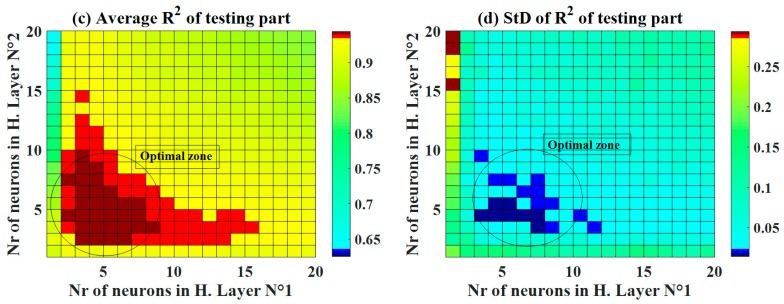
Maps of the performance indicator R^2^ as a function of the number of neurons in hidden layers No. 1 and 2, for both the mean and standard deviation values over 1000 random samplings

Various structures of C-ANN were investigated, as shown in Figure 4, for both the training and testing datasets. With respect to the training dataset, it was observed that higher number of neurons in both hidden layers produced a higher accuracy (R^2^ > 0.95). Differently, an optimal zone was detected to achieve the highest accuracy (R^2^ around 0.95) for the testing dataset (Figure 4c). This is an excellent example to demonstrate that an appropriate ANN structure must be determined before performing ML simulations. To further investigate the effect of neurons in the two hidden layers, standard deviation plots of the R^2^ is presented (Figure 4b,d). The training part exhibited a small standard deviation, in general, with a low number of neurons in the second hidden layer (i.e., from 4 to 8 neurons). On the other hand, an optimal zone was observed for values of standard deviation of R^2^ for the testing dataset. Such a zone was in a similar position with the average values of R^2^, ranging from 3 to 8 neurons in the first hidden layer and from 4 to 6 in the second hidden layer.

**Figure 5:**
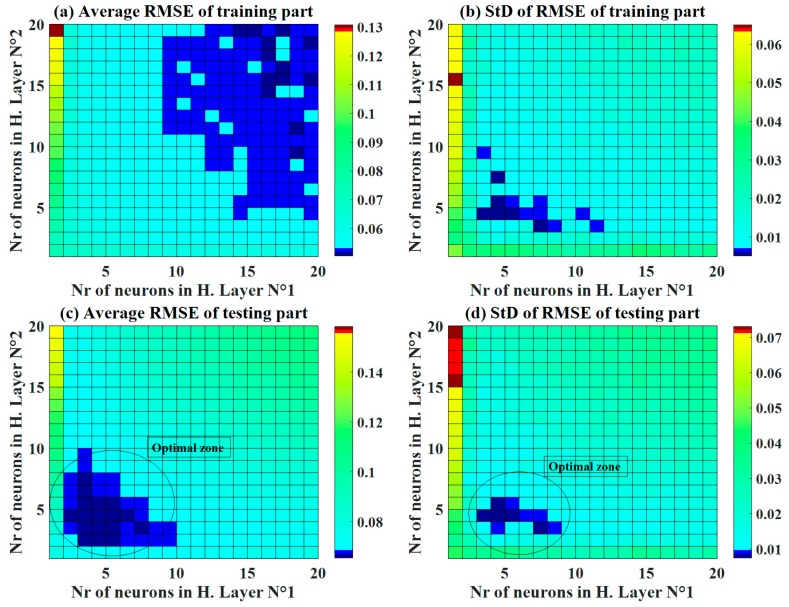
Maps of the performance indicator RMSE as a function of the number of neurons in hidden layers No. 1 and 2, for both the mean and standard deviation values over 1000 random samplings

**Figure 6:**
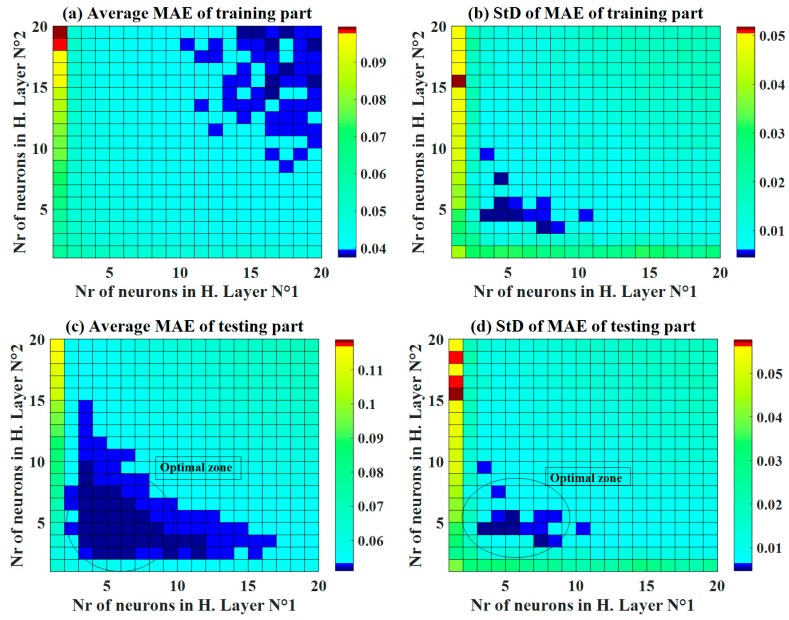
Maps of the performance indicator MAE as a function of the number of neurons in hidden layers No. 1 and 2, for both the mean and standard deviation value over 1000 random samplings (**a–d**).

**Figure 7:**
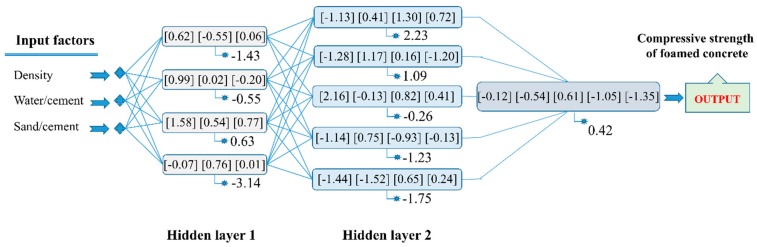
The architecture of the optimal C-ANN-[3–4–5–1] with weights (presented in […]) and biases (presented along the stars).

Using RMSE, Figure 5 displays the average values of RMSE and the corresponding standard deviation for the training and testing datasets. Similar to the case of R^2^, the better the accuracy of C-ANN increased when the number of neurons in the two hidden layers increased. The optimal zone of neurons was also determined with a moderate number of neurons (Figure 5c) for the testing dataset with respect to both the average RMSE values and the standard deviation. It is also noticed that for the training part, the lowest values of standard deviation were found around the C-ANN-[3–4–5–1] zone. Similar observations were found using the average values and standard deviation of MAE (Figure 6). It can be stated that an increase in the number of neurons in the hidden layers can improve the accuracy of the C-ANN for the training dataset, and the optimal architecture of the C-ANN was selected as C-ANN-[3–4–5–1] (Figure 7). 

### 4.3. Robustness of Optimal C-ANN Structure 

**Figure 8:**
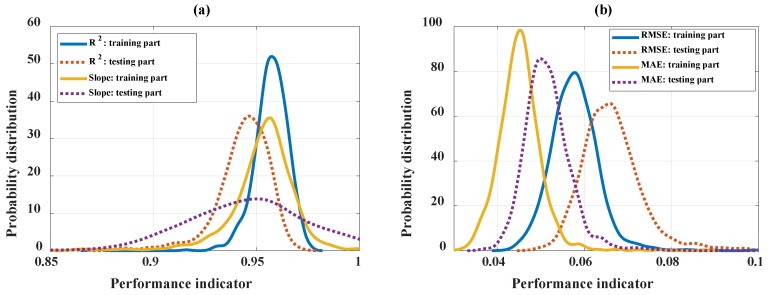
The distribution of performance indicators over 1000 runs using the optimal C-ANN architecture: (**a**) for R^2^ and slope, (**b**) for RMSE and MAE (**a–b**).

The robustness of the optimal C-ANN structure of C-ANN-[3–4–5–1] was analyzed using MCS (Figure 8). From a statistical point of view, Figure 8 shows the distribution of the performance indicators over 1000 realizations using the optimal C-ANN architecture, whereas the statistical analysis is highlighted in Table 3. The values of the average and standard deviation of the R^2^ values are given in Figure 8a and those of the RMSE and MAE are plotted in Figure 8b for the training and testing datasets. 

It can be seen that the performance indicators of the training part is better than the testing one, which could avoid overfitting problems. While the curves of R^2^, RMSE, and MAE were rather narrow, the curves of slope for the testing part were rather broad. In all the cases, the distributions of the results followed a Gaussian distribution but with different standard deviations. In addition, the values of three levels of quantiles (Q25, Q50, and Q75) with the coefficient of variation are given in Table 3. As expected, the optimal architecture provided a high performance of prediction as well as a low value of coefficient of variation (CV). In terms of CV, the model also proved as a robust predictor. Values of CV of RMSE and MAE were approximately 10% due to the small mean values. In case of R^2^ and slope, CVs were lower than 3.2%.

### 4.4. Interpretation of Relationship between of Inputs and Output Using PDP

**Figure 9:**
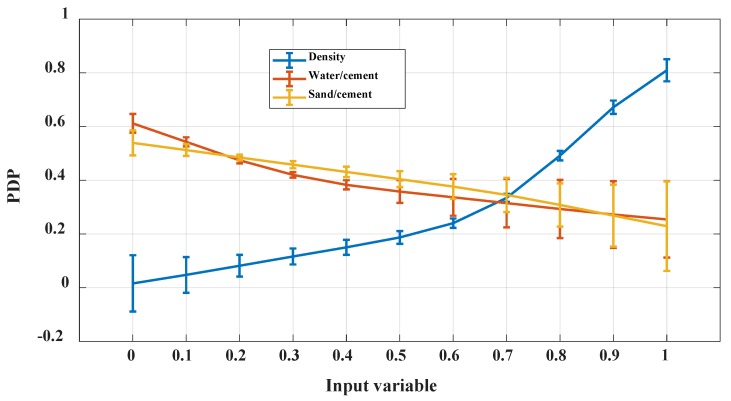
PDP curves in function of input variables.

In general, the use of PDP is effective in interpreting the relationships between the inputs and the predicted output [79]. As seen in Figure 9, all considered input variables affected the 28-days CS of FC; however, at different amplitudes. For instance, the CS of FC decreased when the water/cement ratio (W/C) and sand/cement ratio (S/C) increased, but it increased when dry density (D) increased. Thus, the water/cement ratio (W/C) and sand/cement ratio (S/C) had a negative effect, whereas dry density had a positive effect on the CS of FS. It is seen that the PDP investigation for each input could be approximated by a different fit (i.e., exponential, quadratic, and linear). Table 4 summarizes the overall values of the PDP investigation, including a classification of the influence of the level of inputs and the negative and positive effects of such input to the predicted output.

## 5. Discussion

A machine learning algorithm like C-ANN has been widely and effectively applied in different fields for solving a lot of real-world problems, including material sciences [80,81]. Although this approach is proved as a promising tool and technique for better performance of the model, the accuracy of this approach depends significantly on the selection of network structures [82,83,84]. In this study, a thorough investigation of ANN structures has been carried out for predicting the 28 days CS of FC, which is one of the most important mechanical properties of FC. In addition, it is well-known that the accuracy of the given machine learning algorithm greatly depends on the sampling strategy to construct the model [85,86]. Therefore, MCS was used in this study to fully analyze the capability of all the C-ANN structures, taking into account such variability of the input space in the training phase of the model. As a result, an accuracy map was given where an optimal structure was defined by statistical analysis of many criteria, such as the maximum average values of the R^2^, RMSE, and MAE, as well as the minimum values of the standard deviation of R^2^, RMSE, and MAE. 

The investigation results showed that a good result could be obtained with simple machine learning algorithms like the C-ANN, as long as the structure and the parameters were well established. The optimization results of the C-ANN structure also showed that increasing the number of neurons in the hidden layers can enhance the accuracy of the C-ANN for the training dataset, and the optimal architecture of the C-ANN was selected as C-ANN-[3–4–5–1]. It is worth noticing that the maximum value of the R2 for the testing part, in this study, could reach an accuracy of R^2^ = 0.972 (R = 0.986). This value is amongst the best accuracies reported in the literature, as compared with results in [25,47]. Similar observations with respect to criteria such as RMSE or MAE were also noticed and compared with previously published works, such as the RMSE = 1.808 [47] or 1.65 [25], but these studies used only 177 instances [25] or 150 samples [47].

In addition, it is also noticed that the performance of machine learning models such as C-ANN depends on the selection of input factors used in the datasets. Therefore, a sensitivity analysis should be carried out to (i) verify the performance of the C-ANN model on the choice of features in the input space and (ii) interpret the relationships between the input variables and the 28 days FC of CS. For this, such relationships were investigated thanks to PDP. In general, PDP analysis is given as the result of one constructed machine learning model. Thus, the results are influenced by the accuracy of such model and it could vary in function of the random sampling strategies. Again, the PDP analysis in this study was taken as the average of 1000 reliable simulations and could be confident for further investigations. The results of this study are in agreement with the findings in the literature, which stated that the 28-days CS of FC increased exponentially with an increase in dry density (D) [23,47]. Besides, a previous study of Hoff [87] indicated that for a certain range of density, this relationship can be linear, as observed for the scaled density from 0 to 0.5 in this study. However, for the whole range of density, an exponential relationship is assumed. The positive effect of dry density (D) and cement content was confirmed and had a significant role in designing FC mixes [47]. Meanwhile, a negative effect on the CS of FC was confirmed to be associated with an increase in the water/cement ratio (W/C) and sand/cement ratio (S/C) [47]. Moreover, the water/cement ratio (W/C) is a controlling factor of the CS of FC [88], or, an appropriate content of water enhances the consistency and stability of FC, which increase the CS of FC [10,89].

## 6. Conclusion

Despite a number of studies on the prediction of the CS of FC using ML, the prediction performance could still benefit from more in-depth investigations. The current study showed a simple but efficient manner to use an ANN structure to predict the 28-days CS of FC. An optimal C-ANN-[3–4–5–1] was proposed, where the robustness was confirmed in the presence of a random dataset splitting over 1000 MCS. Statistical assessment of the results was derived to verify the reliability of predicted results and testify the convergence of the outcomes of C-ANN. A simple and optimal C-ANN structure was proven to produce comparative results with complex algorithms proposed in the literature, where the value of R^2^ could reach R^2^ = 0.972. A comprehensive interpretation of the results using PDP investigations was performed and derived several relationships, revealing the dependence of the 28-days CS of FC on the dry density (D), water/cement ratio (W/C), and sand/cement ratio (S/C). Overall, dry density (D) was found to be the most affecting factor, which had a significant role on the 28-days CS of FC. The results of the present study could facilitate and enhance the use of C-ANN in other civil engineering-related problems. Two perspectives can be envisioned: (i) gathering more data to cover larger ranges of input and output variables, and (ii) implementing optimization techniques to improve the accuracy of the C-ANN algorithm.

## Figures and Tables

**Table 1:** Database collected in this study.

References	No. of Samples	Proportion (%)
Abd et al. (2017) [47]	144	65.45
Asadzadeh and Khoshbayan (2018) [21]	24	10.91
Hilal et al. (2015) [3]	7	3.18
Jones and McCarthy (2005) [12]	12	5.45
Kozłowski et al. (2015) [17]	4	1.82
Mounanga et al. (2008) [16]	4	1.82
Richard and Ramli (2013) [50]	1	0.45
Pan et al. (2007) [6]	12	5.45
Tam et al. (1987) [10]	9	4.09
Tikalsky et al. (2004) [11]	3	1.38
**Total**	**220**	**100**

**Table 2:** Statistical analysis of the data used in this study.

	Dry Density	Water/Cement	Sand/Cement	Compressive Strength (28 days)
Notation	D	W/C	S/C	CS
Unit	(kg/m^3^)	-	-	(MPa)
Role	Input	Input	Input	Output
Min	430.00	0.26	0.00	0.60
Average	1566.33	0.44	1.20	22.94
Median	1639.50	0.40	1.00	24.50
Max	2009.48	0.83	4.29	48.88
SD	369.49	0.12	0.67	13.42
CV (%)	23.59	0.28	0.56	0.59

**Table 3:** Statistical analysis of performance indicators over 1000 runs using the optimal C-ANN architecture.

Parameters	Training Part				Testing Part			
	R^2^	Slope	RMSE	MAE	R^2^	Slope	RMSE	MAE
Min	0.871	0.779	0.043	0.031	0.800	0.776	0.049	0.037
Q25	0.953	0.947	0.054	0.042	0.938	0.925	0.062	0.048
Q50	0.958	0.955	0.058	0.045	0.945	0.947	0.066	0.051
Q75	0.963	0.962	0.061	0.048	0.952	0.964	0.070	0.054
Max	0.976	1.026	0.109	0.088	0.972	1.027	0.140	0.114
Mean	0.957	0.953	0.058	0.045	0.943	0.945	0.067	0.052
SD*	0.009	0.019	0.006	0.005	0.015	0.031	0.008	0.006
CV (%)	0.907	1.987	9.880	10.874	1.594	3.267	11.718	10.785

SD* = standard deviation.

**Table 4:** Analysis and interpretation of PDP curves.

Input	Fit	Equation Form	Effect	Nature of Correlation Effect	Rank
Density	Exponential	CS = 0.044* exp(2.967 D)	Positive	Nonlinear	1
W/C	Quadratic	CS = 0.31 W/C ^2^ - 0.64 W/C + 0.60	Negative	Nonlinear	3
S/C	Linear	CS = -0.3 S/C + 0.55	Negative	Linear	2
